# People and performance, cardiac clinic maturity – measurable indicator based on Medinet Heart Center, Wroclaw, Poland. Ten years medical history achievements

**DOI:** 10.1186/1749-8090-8-S1-O91

**Published:** 2013-09-11

**Authors:** C Augustyn, H Kociemska, R Jadach, R Cichon

**Affiliations:** 1 Medinet Heart Center, Wroclaw, Poland; 2 University of Economics, Wroclaw, Poland; 3 Medical University, Warsaw, Poland

## Background

Development of a new cardiac surgery clinic should be combination of increase in number of cardiac procedures with decrease in number of complications.

## Methods

Descriptive analysis. Qualitative and quantitative empirical data analysis medical clinic results from 2002-2012, human resources department data analysis from 2002-2012.

## Results

Along with increased number of complex surgery (such as aortic diseases, combined coronary and valves procedures, congenital diseases) presenting the higher risk combined with the more complicated preoperative patient state, do not increases both: number of deaths nor postoperative complications, caused by improving preoperative diagnosis and more experienced intraoperative judgement as well as increasing medical personnel qualifications. It’s worth to present as an example: starting from 2002, total no. of operation 457 with mortality rate of 6,7% , through 878 operation in 2012 and mortality rate 3,4% performed by 9 surgeons 2002 and 11 surgeons in 2012. Cardiac ward maturity might be recognized due to surgeon’s scientific development parameters (such as doctors degree, medical boards certifications, habilitations etc) coexisting with: low operational staff rotation, increasing number of operation attributable to single surgeon, decreasing number of deaths or complications.

## Conclusions

by providing the stable operational teams with increasing number of operations and possibilities of scientific development, there is a significant improvement in clinical results, increase of patients and personnel satisfactions.

